# Ring vaccination of COVID‐19 vaccines in medium‐ and high‐risk areas of countries with low incidence of SARS‐CoV‐2 infection

**DOI:** 10.1002/ctm2.331

**Published:** 2021-02-17

**Authors:** Wei Xu, Shan Su, Shibo Jiang

**Affiliations:** ^1^ Key Laboratory of Medical Molecular Virology (MOE/MOH/CAM) School of Basic Medical Sciences Shanghai Institute of Infectious Disease and Biosecurity Fudan University Shanghai China

Typically, winter brings on the peak months of respiratory infections. Indeed, colder weather in the Northern Hemisphere has brought multiple new waves of coronavirus infectious disease 2019 (COVID‐19) epidemic to some countries where COVID‐19 epidemic was under control before. For example, since the beginning of winter, several small‐scale outbreaks of COVID‐19 have been caused by local or imported cases in China. Meanwhile, the emergence of viral variants with higher transmissibility and the possible spread of severe acute respiratory syndrome coronavirus 2 (SARS‐CoV‐2) through logistic channels have caused further alarm among researchers and health care workers. It is, however, good news that a number of COVID‐19 vaccines have been approved for general or emergency use and are being used for inoculation globally. On the other hand, the capacity to produce COVID‐19 vaccines is limited, making it necessary to vaccinate in batches to gradually achieve herd immunity. Many countries, such as the United States, the United Kingdom, and Germany, began their vaccination programs with the intention of prioritizing health care workers and the elderly, as the former are at higher risk of infection and the latter are at higher risk for mortality.^1^

Here, we suggest that ring vaccination be adopted to supplement the large‐scale SARS‐CoV‐2 nucleic acid detection strategy in the medium‐ and high‐risk areas in countries with relatively low incidence because some infected persons may not be detected because of the false‐negative results and some uninfected individuals may become infected later when they return to work in the contaminated environment that has not been fully cleaned.

Ring vaccination, which controls an outbreak by vaccinating and monitoring the contacts of confirmed infected persons, has contributed to the eradication of smallpox.^2^ This strategy also played a significant role in controlling the spread of Ebola virus and assessing the efficacy of vaccine candidates during the Ebola epidemic in 2017.^3^ In order to control the COVID‐19 epidemic, our concept of ring vaccination strategy is comprised of “two rings” (Figure 1). The first ring consists of close contacts of confirmed SARS‐CoV‐2‐infected individuals, or those exposed to a SARS‐CoV‐2‐infected person in the proximate environment, such as the passengers in the same compartment. The second ring consists of close contacts of, or those exposed to, people from the first ring. Through this two‐ring strategy, the incidence of new SARS‐CoV‐2‐infected cases would be reduced because the herd immunity among this high‐risk population could be established, thus the risk of further transmission of SARS‐CoV‐2 to a broader population would be eliminated or reduced. Furthermore, these vaccinated populations even if they return to work in the contaminated environment would be protected from SARS‐CoV‐2 infection in the future.

**FIGURE 1:**
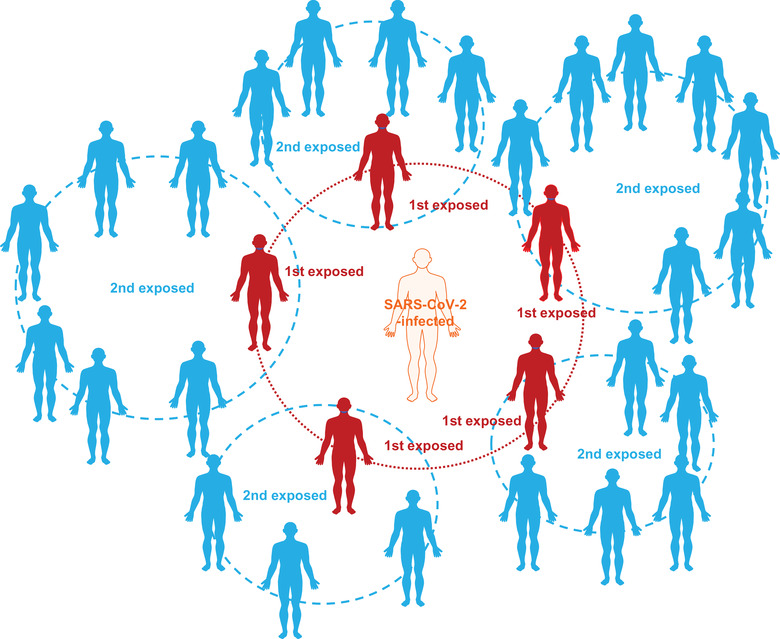
Schematic diagram of ring vaccination strategy. The first ring (red) consists of close contacts of confirmed severe acute respiratory syndrome coronavirus 2 (SARS‐CoV‐2)‐infected individuals, or those exposed to a SARS‐CoV‐2‐infected person in the proximate environment, such as the passengers in the same compartment. The second ring (blue) consists of close contacts of, or those exposed to, people from the first ring. People in these two rings should be vaccinated with priority

It should be noted that ring vaccination is not a universal strategy for all viral outbreaks. A numerical analysis of the effectiveness of ring vaccination estimated that this strategy could successfully contain an outbreak for values of effective reproduction up to 1.6. Therefore, this strategy would be feasible for viruses having lower transmissibility or viruses with high transmissibility, but under effective control.^4^ To explain, despite the rapid spread of SARS‐CoV‐2 worldwide, the COVID‐19 epidemic is still well controlled in some countries, such as China, Australia, Norway, and many other countries in Asia.^5^ In these countries, the newly confirmed cases are scattered, and, more importantly, the disease surveillance network is timely, efficient, and well organized. Therefore, the epidemiological data of SARS‐CoV‐2‐infected persons, including contact history, are well documented. The above information, as well as the advanced information network for the notification and vaccination in these countries can guarantee the implementation of the ring vaccination strategy. Thus, for these countries with relatively low morbidity, ring vaccination would be a good supplement for the current large‐scale vaccination programs to control COVID‐19 epidemic, even with limited vaccine production.

## AUTHOR CONTRIBUTIONS

Wei Xu and Shan Su drafted the manuscript. Shibo Jiang conceived the study idea and modified the draft.
